# Identification of a Novel Biomarker for Biliary Tract Cancer Using Matrix-Assisted Laser Desorption/Ionization Time-of-Flight Mass Spectrometry

**DOI:** 10.1155/2012/108609

**Published:** 2012-07-25

**Authors:** Shintaro Kikkawa, Kazuyuki Sogawa, Mamoru Satoh, Hiroshi Umemura, Yoshio Kodera, Kazuyuki Matsushita, Takeshi Tomonaga, Masaru Miyazaki, Osamu Yokosuka, Fumio Nomura

**Affiliations:** ^1^Department of Medicine and Clinical Oncology, Graduate School of Medicine, Chiba University, 1-8-1 Inohana, Chuo-ku, Chiba, Chiba City 260-8670, Japan; ^2^Clinical Proteomics Center, Chiba University Hospital, 1-8-1 Inohana, Chuo-ku, Chiba, Chiba City 260-8670, Japan; ^3^Department of Molecular Diagnosis, Graduate School of Medicine, Chiba University, 1-8-1 Inohana, Chuo-ku, Chiba, Chiba City 260-8670, Japan; ^4^Department of Physics, School of Science, Kitasato University, 1-15-1 Kitasato, Minami-ku, Kanagawa, Sagamihara City 228-8555, Japan; ^5^Laboratory of Proteome Research, National Institute of Biomedical Innovation, 7-6-8 Saito Asagi, Osaka, Ibaraki City 567-0085, Japan; ^6^Department of General Surgery, Graduate School of Medicine, Chiba University, 1-8-1 Inohana, Chuo-ku, Chiba, Chiba City 260-8670, Japan

## Abstract

Early diagnosis of biliary tract cancer (BTC) is important for curative surgical resection. Current tumor markers of BTC are unsatisfactory in terms of sensitivity and specificity. In a search for novel biomarkers for BTC, serum samples obtained from 62 patients with BTC were compared with those from patients with benign biliary diseases and from healthy controls, using the MALDI-TOF/TOF ClinProt system. Initial screening and further validation identified a peak at 4204 Da with significantly greater intensity in the BTC samples. The 4204 Da peak was partially purified and identified as a fragment of prothrombin by amino acid sequencing. The sensitivity of the 4204 Da peptide for detection of stage I BTC cancer was greater than those for CEA and CA19-9. Also, serum levels of the 4204 Da peptide were above the cut-off level in 15 (79%) of 19 cases in which the CEA and CA19-9 levels were both within their cut-off values. Receiver operating characteristic analysis showed that the combination of the 4204 Da peptide and CA19-9 was significantly more sensitive for detection of stage I BTC cancer compared to CEA and CA19-9. These results suggest that this protein fragment may be a promising biomarker for biliary tract cancer.

## 1. Introduction

Biliary tract cancer (BTC) is a neoplasm that accounts for 3% of all gastrointestinal cancers and 15% of all primary liver cancers. Over the last two decades, the incidence of BTC has risen, mainly due to an increase in the intrahepatic form [1, 2], which has a particularly high incidence in Northern Thailand [3]. Surgical resection is the only curative treatment and this requires an early diagnosis. Even in cases in which surgical resection with negative histological margins is achieved, the 5-year survival rates range from 20% to 40% [4, 5]. The mean one-year survival rate for unresectable cases is only 6 months [4]. Therefore, there is a need to establish a tool for early diagnosis of BTC. Currently, diagnosis of BTC depends on imaging of the biliary tree using computed tomography (CT), ultrasonography, and endoscopic retrograde cholangiography (ERC) in symptomatic subjects. Brush cytology during ERC can lead to morphological diagnosis, but the sensitivity is limited because of the highly desmoplastic reaction of BTC [5, 6]. For these reasons, tumor markers that can detect BTC with high diagnostic efficiency are urgently needed. Carcinoembryonic antigen (CEA) and carbohydrate antigen 19.9 (CA19-9) are tumor markers that are used for diagnosis of BTC, but their sensitivity and specificity are unsatisfactory [2, 7]. 

Proteome analysis is increasingly being applied to cancer biomarker discovery. Surface enhanced laser desorption/ionization time-of-flight mass spectrometry (SELDI-TOF MS) is a proteomics technique used for high-throughput fingerprinting of serum proteins [8]. We have used this technology to identify diagnostic markers for alcohol abuse [9] and a prognostic marker for pancreatic cancer [10]. SELDI-TOF MS can be used to analyze many samples rapidly and simultaneously, but has drawbacks of high cost and difficulty with protein identification. More recently, high-throughput workflow with matrix-assisted laser desorption/ionization-time of flight/time of flight-mass spectrometry (MALDI-TOF/TOF MS) has been established for discovery and identification of serum peptides [11]. This method uses magnetic beads with different chemical chromatographic surfaces, instead of ProteinChip arrays. Proteins selectively bound to the magnetic beads are eluted and analyzed by MALDI-TOF/TOF MS. Compared with the SELDI-TOF MS ProteinChip system, the cost is low, and subsequent protein identification is relatively easy. We recently used the ClinProt system for MALDI-TOF/TOF MS to detect novel biomarkers for alcohol abuse that could not be detected using SELDI [12]. In the present study, we carried out a serum peptidome study to identify novel biomarkers for biliary tract cancer using the MALDI-TOF/TOF MS ClinProt system.

## 2. Methods

### 2.1. Patients and Samples

Serum samples were obtained from 62 patients with BTC (36 males, 26 females; median age 64.7 years old, range 27–81 years old), 30 age-matched healthy controls (18 males, 12 females; median age 65.5 years old, range 61–69 years old), and 30 age-matched patients with benign biliary disease (18 males, 12 females; median age 64.4 years old, range 27–90 years old). Clinicopathological data for all the subjects are shown in Tables 1, 2, and 3. The BTC group (Table 2) included cases of intrahepatic cholangiocarcinoma (*n* = 17), Klatskin tumor (*n* = 7), extrahepatic cholangiocarcinoma (*n* = 16), tumor of the ampulla of Vater (*n* = 6), and gallbladder tumor (*n* = 16). The pathological stages of the BTC patients were defined according to the Union Internationale Contre le Cancer tumor node metastasis classification [13]. The patients with benign biliary diseases (Table 3) included cases of cholelithiasis (*n* = 24), benign fibrous stricture (*n* = 4), and primary sclerosing cholangitis (*n* = 2). All the cases of BTC were diagnosed by radiological imaging. In 58 cases, cytology was also compatible with the diagnosis. All of the patients with benign biliary disease were diagnosed by endoscopic retrograde cholangiopancreatography and were followed up for more than 12 months to confirm that they had no malignancy. Serum samples were obtained and processed under standardized conditions that we have described elsewhere [14] and were stored at −80°C until analysis. Written informed consent was obtained from all the subjects. The study was approved by the Ethics Committee of Chiba University School of Medicine.

### 2.2. Serum Pretreatment with Magnetic Beads Using the ClinProt Robot

We used weak cation exchange (WCX) magnetic beads (Bruker Daltonics) and performed serum peptidome fractionation according to the manufacturer's protocol. A 5 *μ*L serum sample was mixed with 10 *μ*L of binding buffer to which 5 *μ*L of WCX beads was added, and the solution was carefully mixed. The peptides in the serum were then allowed to bind to the WCX beads for 5 min. The tube was then placed in a magnetic bead separator (Bruker Daltonics) for separating unbound beads, and the supernatant was removed. The beads were washed three times with 100 *μ*L of washing buffer, and the proteins as well as peptides were then eluted from the magnetic beads with 10 *μ*L each of elution and stabilization buffer. Thereafter, 2 *μ*L of peptide elution solution was mixed with 20 *μ*L of alpha-cyano-4-hydroxycinnamic acid matrix (Bruker Daltonics). Then 0.8 *μ*L of this mixture was spotted onto an AnchorChip target plate (Bruker Daltonics) and crystallized. Each sample was duplicated, and quadruplicate spotting was performed using each eluate; eight spots were developed from each sample. The mean spectra from these eight spots were used for data analyses. These procedures from bead fractionation to spotting were performed automatically using the ClinProt robot (Bruker Daltonics) under strictly controlled humidity, as we previously described [14].

### 2.3. Mass Spectrometry

The AnchorChip target plate was placed in an AutoFlex II TOF/TOF mass spectrometer (Bruker Daltonics) controlled by Flexcontrol 2.4 software (Bruker Daltonics). The instrument was equipped with a 337 nm nitrogen laser, delayed-extraction electronics, and a 25 Hz digitizer. All acquisitions were generated by an automated method included in the instrument software and based on averaging of 1000 randomized shots. Spectra were acquired in positive linear mode in the mass range of 600–10000 Da. Peak clusters were completed using second pass peak sections (signal to noise ratio >  5). The relative peak intensities of *m/z* between 600 and 10000 normalized to a total ion current were expressed in arbitrary units. Calibration was performed using Peptide Calibration Standard II (Bruker Daltonics). All MALDI-TOF MS spectra from *m/z* 1000 to 10000 were analyzed with FlexAnalysis 2.1 and ClinProtools 2.1 software (Bruker Daltonics). 

### 2.4. Protein Identification

A CM ceramic Hyper DF spin column (Bio-Rad Laboratories, Irvine, CA, USA) was washed 3 times with 400 *μ*L of MB-WCX binding solution (Bruker Daltonics). Serum samples (320 *μ*L) were diluted 5-fold with binding buffer and the diluted sample (1600 *μ*L) was applied to the spin column. The sample was allowed to bind at 4°C for 1 h on a shaker and then the spin column was washed 3 times with 400 *μ*L of binding buffer. Finally, 320 *μ*L of MB-WCX stabilization solution (Bruker Daltonics) was added to the spin column for elution. Ten volumes of ice cold acetone were added to the eluate. Peptides/proteins were allowed to precipitate at −20°C for 2 h and then obtained by centrifugation at 13000 g for 10 min at 4°C. After decanting the acetone, the peptides/proteins were allowed to air dry. The dried pellets were resuspended in buffer (0.1% trifluoroacetic acid in water, vol/vol) and further separated by reversed-phase HPLC in an automated HPLC system (Shiseido Nanospace SI-2, Shiseido Fine Chemicals, Tokyo, Japan). The concentrated flow-through sample (75 *μ*L) was directly loaded onto an Intrada WP-RP column (Imtakt, Kyoto, Japan). The reversed-phase separations for each flow-through fraction were performed using a multisegment elution gradient with eluent A (0.1% trifluoroacetic acid in water, vol/vol) and eluent B (0.08% trifluoroacetic acid in 90% acetonitrile, vol/vol). The gradient elution program consisted of three steps with increasing concentrations of eluent B (5% B for 5 min, 5% to 95% B for 23 min, and 95% B for 11 min) followed by 5% B for 21 min for reequilibration of the column at a flow rate of 0.40 mL/min for a total run time of 60 min. Based on the chromatogram recorded by measuring the absorbance of the eluate at 280 nm, fractions eluted at retention times between 19.1 and 39.1 min were collected in 40 0.2 mL aliquots at a fraction size setting of 0.5 min. Fractions including objective peaks were confirmed by MALDI-TOF MS. N-terminal amino acid sequence analysis was performed using a Procise 494 cLC protein sequencing system (Applied Biosystems, Foster City, CA, USA).

### 2.5. Statistical Analysis

Univariate analysis of individual peaks was performed using a nonparametric Mann-Whitney *U* test, with *P* < 0.05 considered significant. Discriminatory power for putative markers was further evaluated by receiver operating characteristic (ROC) analysis and the area under the curve (AUC) using IBM SPSS Statistics 18 (SPSS Inc., Ill, USA).

## 3. Results

### 3.1. MALDI-TOF-MS Analysis of Peptides in BTC Sera

**Figure 1:**
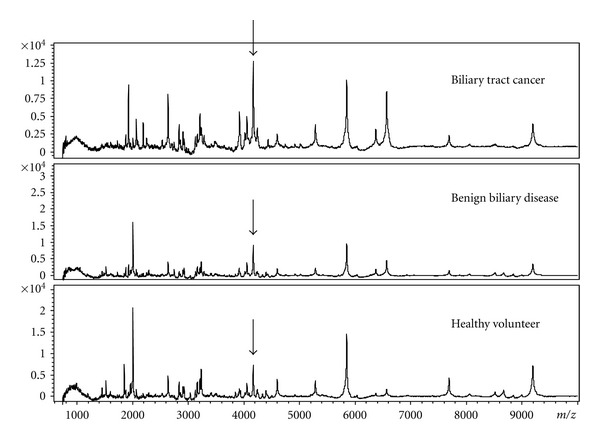
The protein mass profile between *m/z* 0 and 10000 highlighting the differentially expressed peaks in serum from healthy volunteers, patients with benign biliary disease, and BTC patients. The *m/z* 4204 peak (indicated by arrows) intensity was higher in cancer patients compared with patients with benign disease and healthy volunteers.

As a first step, we compared the peptide profiles of serum samples obtained from BTC patients (*n* = 30) with those from healthy controls (*n* = 12) as a training set (Table 4). Totally 134 peaks were detected and compared in the MALDI proteomic profile. Total of 22 peak intensities differed significantly between the BTC patients and healthy controls, including 12 that were higher and 10 that were lower in the BTC group. In typical spectra for serum samples from each group (Figure 1), the intensity of the 4204 *m/z* peak was higher in BTC samples compared with those from patients with benign biliary diseases and from healthy controls.

**Figure 2:**
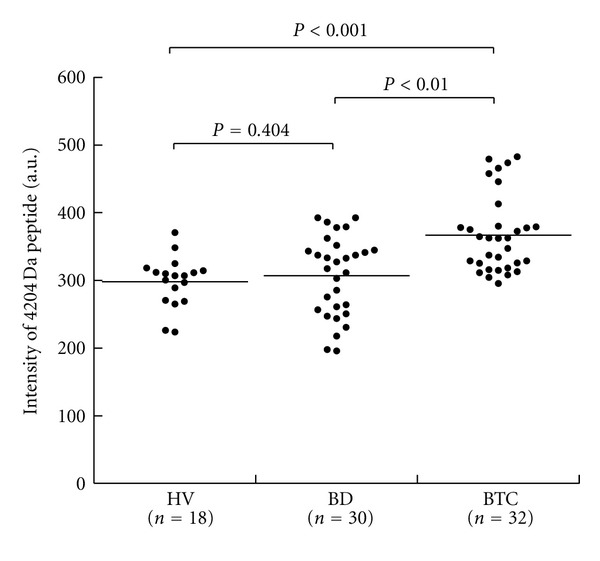
Normalized intensities of the peak corresponding to the 4204 Da peptide in serum from healthy volunteers (*n* = 18), patients with benign biliary disease (*n* = 30), and BTC patients (*n* = 32). The peak intensity was significantly higher in sera obtained from patients with biliary tract cancer (BTC) compared with sera from healthy volunteers. There was no significant difference in intensity between BTC sera and benign biliary disease (BB) sera (Mann-Whitney *U* test).

Next, we tested whether the differences observed in the 22 peaks in the training set were reproducible in another set of samples (test set) (Table 5). 32 BTC patients, 30 benign biliary patients, and 18 healthy controls were included in the test set. Out of these 22 peaks, the intensities of 2 peaks (3272 *m/z* and 4204 *m/z*) were again significantly different between the BTC and control groups. Out of these 2 peaks, the intensity of one peak (4204 *m/z*) was also found to be significantly higher in the BTC group compared to the benign disease group. The relative intensities of the 4204 *m/z* peak in sera obtained from the three groups of subjects are summarized in Figure 2.

### 3.2. Identification of the 4204 Da Peptide as a Fragment of Prothrombin

**Figure 3:**
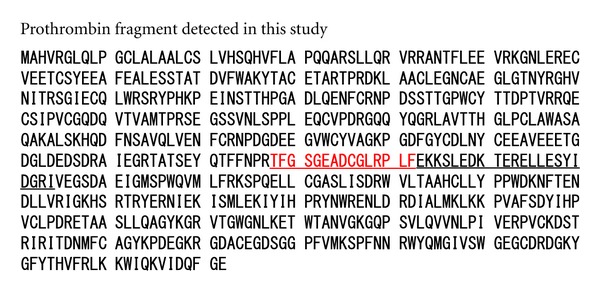
N-terminal amino acid sequence of the purified fraction. Red letters identify the N-terminal sequence. The molecular weight of the underlined region is 4204 Da.

Partial purification of the peptide corresponding to the 4204 *m/z* peak was conducted as outlined in Section 2. N-terminal amino acid sequencing of trypsin digests of the final preparation containing the 4204 Da peptide revealed that it was a fragment of prothrombin (Figure 3).

### 3.3. Diagnostic Value of the 4204 Da Peptide Compared with Conventional Markers

**Figure 4:**
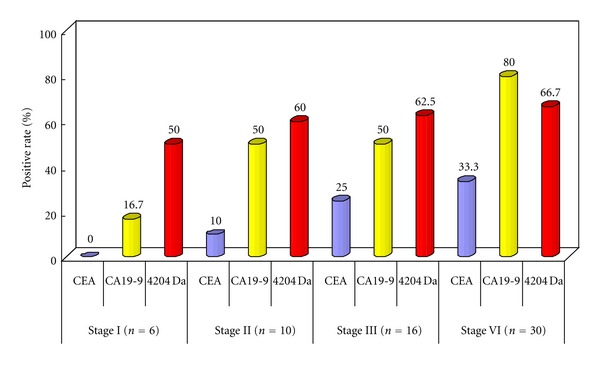
Positive rates of detection of the 4204 Da peptide, CEA, and CA19-9 in each UICC stage.

Patients with BTC were divided into 4 groups based on clinical stage. The sensitivities of CEA, CA19-9, and 4204 Da in the BTC patients were determined (Figure 4). The optimal cut-off point for CEA, CA19-9, and the 4204 Da peptide were selected based on mean + 2SD in healthy subjects. The cut-off levels for CEA, CA19-9, and the 4204 Da peptide were set at 6.4 ng/mL, 33.5 U/mL, and 372.1 AU, respectively. The sensitivities of CEA, CA19-9, and the 4204 Da peptide in stage IV patients were 33.3%, 80.0%, and 66.7%, and the specificities of CEA, CA19-9, and the 4204 Da peptide were 93.3%, 93.3%, 96.7%, respectively. In contrast, these sensitivities in stage I patients were 0.0%, 16.7%, and 50.0%, and these specificities were 93.3%, 93.3%, 96.7%. The sensitivity of the 4204 Da peptide was also greater than those of CEA and CA19-9 in stage II patients.

**Figure 5:**
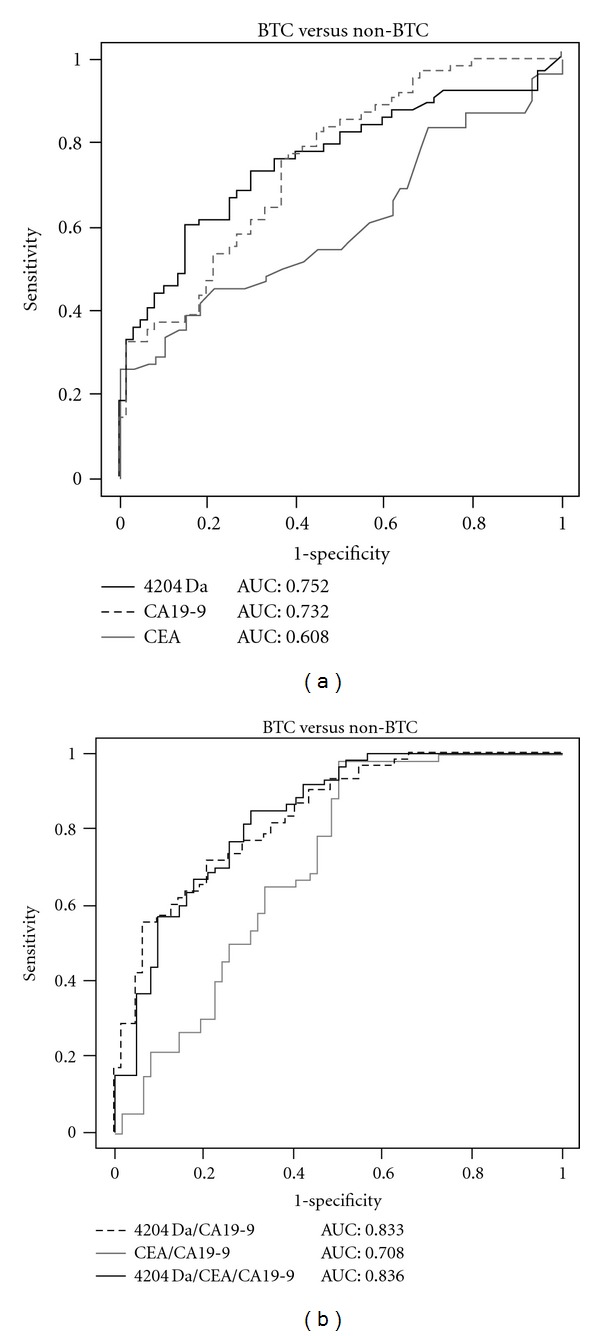
ROC analyses of the performance of the 4204 Da peptide, CA19-9, and CEA. (a) AUC was 0.752 for 4204 Da, 0.732 for CA19-9, and 0.608 for CEA. (b) AUC was 0.833 for 4204 Da + CA19-9, 0.708 for CA19-9 + CEA, and 0.836 for 4204 Da + CEA + CA19-9.

The ROC curves for the 4204 Da peptide, CEA, and CA19-9 as single markers and combinations are shown in Figure 5. The sensitivities were determined from the results for the 62 patients with BTC and specificities were based on the 60 non-BTC subjects. The AUCs for the 4204 Da peptide, CEA, and CA19-9 as single markers were 0.75, 0.60, and 0.732, respectively. The AUC for the combination of the 4204 Da peptide and CA19-9 was significantly greater than that for CEA and CA19-9 (*P* < 0.01). The sensitivity and specificity for combination of the 4204 Da and CA19-9 were 59.8% and 84.0%.

The 62 patients with BTC were also classified into 8 groups based on their tumor marker status, as shown in Table 6. The cut-off values for CEA and CA19-9 were set at 5 ng/mL and 37 U/mL, respectively. The optimal cut-off point for the 4204 Da peptide was selected based on the ROC analysis. The 4204 Da peptide level was greater than the cut-off value in 15 (79%) of 19 cases in which the CEA and CA19-9 levels were within their respective cut-off values.

## 4. Discussion

The sequencing of the human genome has opened the door for comprehensive analysis of all mRNAs (transcriptome) and proteins (proteome). However, the levels of mRNAs are not necessarily predictive of the corresponding protein levels. Indeed, a recent report indicated that the consistency between cDNA microarray and proteome-based profiles is limited for identification of candidate biomarkers in renal cell carcinoma [15]. Therefore, proteome analysis is a prerequisite for identification of novel biomarkers.

Biliary tract cancer is a particularly lethal malignancy with a mean 1-year survival of only 6% for unresectable cases [4]. The lack of a sensitive and specific biomarker for early detection of BTC is one of the reasons for this limited survival. Cholangiocarcinoma often grows along the bile duct without forming a mass, and thus is often missed in CT and ultrasound. Serum biomarkers with satisfactory sensitivity and specificity are likely to be beneficial in the clinical management of this malignancy. There have been previous attempts to discover biomarkers for cholangiocarcinoma. Scarlett et al. conducted proteomic profiling of sera from cases of cholangiocarcinoma using SELDI-TOF MS and found that a serum peptide corresponding to a 4463 *m/z* peak had superior discriminatory ability to CA19-9 and CEA, but did not identify the peak [16]. More recently, a membrane protein enrichment strategy coupled with ^18^O labeling-based quantitative proteomics was used to identify proteins that are highly expressed in cholangiocarcinoma tissues [17]. Golgi membrane protein, annexin IV, and epidermal growth factor were proposed as candidate markers. However, their diagnostic roles at the serum level were not described. 

CEA and CA19-9 are tumor markers for BTC with average sensitivity and specificity for detecting cholangiocarcinoma of 51% and 88%, respectively, for CEA, and 71% and 78%, respectively, for CA19-9 [7]. In the present study, the sensitivities of CEA, CA19-9, and the 4204 Da peptide for detection of all BTC cases were 50%, 61.3%, and 75.8%, respectively. It was of note that the sensitivity of 4204 Da in stages I and II patients was far greater than those of the conventional markers and that serum 4204 Da peptide levels were elevated in 79% of cases in which both CA19-9 and CEA were within their reference intervals. These findings suggest that this novel peptide is complementary to conventional markers in diagnosis of BTC. This is supported by the greater AUC with the combination of CEA, CA19-9, and the 4204 Da peptide, compared to individual AUCs.

The result obtained in identification of the 4204 Da peptide was unexpected. The peptide was identified as a fragment of prothrombin, which makes it unlikely that the fragment originated from cancer tissues. It is possible that production of the fragment occurred in the cancer-tissue microenvironment. Alternatively, the 4204 Da peptide might have been generated ex vivo by undefined degradative proteases during the clotting process [18]. The exact mechanism for production of the 4204 Da peptide remains to be clarified. We also note that intrahepatic cholangiocellular carcinoma, extrahepatic cholangiocellular carcinoma, and gall bladder carcinoma were analyzed together as BTCs in the present study. Separate analyses of these diseases on a larger scale are needed to discover biomarkers that are more specific for each form of BTC. Also, antibody-based verification will be necessary to further confirm the findings obtained in this study. Bile samples may also be an alternative for discovery of disease markers leaking from the biliary tree [19].

## Figures and Tables

**Table 1:** Clinical characteristics of patients with biliary tract cancer or benign biliary disease and healthy volunteers.

Healthy volunteers	Benign biliary disease	Biliary tract cancer	
No. of patients	30	30	62
Male/female	18/12	18/12	36/26
Mean age	65.5 ± 4.5	64.4 ± 39.0	64.7 ± 37.4
CEA (ng/mL)	3.1 ± 3.5	2.5 ± 3.6	37.6 ± 1577.3
CA19-9 (U/mL)	15.3 ± 18.6	70.4 ± 603.9	5033.6 ± 190367.2

**Table 2:** Characteristics of patients with biliary tract cancer.

Item	Number of patients
Location	
Extrahepatic	16
Intrahepatic	17
Klatskin	7
Ampulla of Vater	6
Gallbladder	16
UICC stage	
Stage I	6
Stage II	10
Stage III	16
Stage IV	30

**Table 3:** Characteristics of patients with benign biliary disease (*n* = 30).

Item	Number of patients
Cholelithiasis	26
Benign fibrous stricture	2
Primary sclerosing cholangitis	2

**Table 4:** Discriminatory peaks and *P* values in the training set.

Higher in biliary tract cancer group		Lower in biliary tract cancer group	
*m/z*	*P* value	*m/z*	*P* value
1207	<0.0001	1944	<0.0001
1466	<0.0001	2669	<0.001
3261	<0.001	2931	<0.0001
3950	<0.001	3239	<0.0001
4202	<0.001	3272	<0.001
4635	<0.001	3878	<0.01
4654	<0.001	4051	<0.001
5791	<0.0001	4086	<0.0001
5890	<0.0001	4276	<0.0001
5929	<0.001	6414	<0.001
9246	<0.001
9285	<0.0001

**Table 5:** Discriminatory peaks detected in the training set and test set.

Higher in biliary tract cancer group		Lower in biliary tract cancer group	
*m/z*	*P* value	*m/z*	*P* value
4202	<0.001	3272	<0.001

**Table 6:** Positive or negative status of 4204 Da peptide, CEA, and CA19-9 in patients with biliary tract cancer.

CEA (≥5 ng/mL)	CA19-9 (≥37 U/mL)	4204 Da peptide (≥322 A.U.)	Number of patients
−	−	−	4
−	−	+	15
−	+	−	6
+	−	−	2
+	+	−	3
+	−	+	2
−	+	+	18
+	+	+	12

## Abbreviations

AU: Arbitrary unit
BTC: Biliary tract cancer
MALDI-TOF MS: Matrix-associated laser desorption ionization time-of-flight mass spectrometry
CEA: Carcinoembryonic antigen
CA19-9: Carbon hydrate antigen 19.9
ROC: Receiver operation characteristic
AUC: Area under the curve.
